# Defying risk: moving from resilience to the capacity to adapt, a contribution to mental health prevention

**DOI:** 10.3389/fpsyg.2025.1600841

**Published:** 2025-07-02

**Authors:** Sasha Rudenstine, Samantha Feliz, Oneysha Brown, Talia Schulder

**Affiliations:** City College of New York (CUNY), New York City, NY, United States

**Keywords:** mental health, adaptability, stressor, resilience, prevention

## Introduction

Life is full of inevitable stressors and their link to psychological distress is indisputable (Smith and Pollak, 2020). Furthermore, historically minoritized populations and/or individuals of lower socioeconomic status have consistently been shown to experience a disproportionate number of lifetime stressors and have higher rates of mental disorders across the life course (Hogg et al., 2022). In the context of these inequities and stressors, enhancing adaptability will equip individuals to offset life's stressors and reduce the incidence of mental disorders (Fiscella and Williams, 2005; Moore et al., 2015; Gruebner et al., 2017; SAMHSA, 2017). To date, focus has been primarily on resilience, defined as recovery after distress *following* a stressor, and on posttraumatic growth, defined as *changes* that occur *as a result of* experiencing stressors, and less so on adaptability, defined as acclimating and maintaining adequate functioning *amidst* persistent and/or recurrent stressors. Therefore unlike resilience, which is a trajectory of health in which an individual returns to baseline psychological functioning following a period of distress in the context of a stressor, and unlike posttraumatic growth, which captures the changes that can occur from experiencing significant challenge, adaptability has the potential to “immunize” individuals against the negative mental health outcomes of persistent stress (Tedeschi et al., 2018; Tugade and Fredrickson, 2004; Ungar, 2013). In this opinion piece, we posit that promoting adaptability at the individual, network, and local/national levels has the potential to mitigate the onset of mental illness and foster overall psychological wellbeing.

Mental disorders constitute a significant global health burden, affecting individuals across diverse age groups, socioeconomic backgrounds, and cultures (National Institute of Mental Health, 2025). The prevalence of mental disorders has been increasing steadily; recent estimates suggest approximately one in four individuals will experience a mental health condition during their lifetime (Johns Hopkins Medicine, 2024). The COVID-19 pandemic exacerbated psychiatric distress and was associated with a global increase in disorders, particularly among children and adolescents (World Health Organization, 2022a,b). Following first onset, one's vulnerability increases for comorbid illnesses and persistent episodes over the life course (Koenen et al., 2013). The consequences of mental disorders extend beyond the individual, affecting families, communities, and societies at large (The Carter Center, 2018; Chan et al., 2023; Jenkins et al., 2011; Mental Health, 2025; Roehrig, 2016; Trautmann et al., 2016). Nonetheless, mental health promotion and prevention efforts have not advanced as much as those for other health conditions such as cardiovascular disease and cancer (Jacka et al., 2013; Fusar-Poli et al., 2019).

## Refining terminology: adaptability and why it matters for prevention

Although resilience is well-studied, there is no singular definition. One frequently used definition is that resilience is a trajectory of health characterized by a brief period of psychological distress after exposure to a stressor, followed by a return to health (Albayrak et al., 2024; Chmitorz et al., 2018; Rudenstine and Galea, 2015). Conceptualizing resilience as a trajectory of health implies that a potentially traumatic occurrence or stressor is a discrete event with a clear beginning and end. However, the nature of stress exposure is rarely so circumscribed. Instead, large swaths of the population experience chronic and cumulative stressors over the life course.

**Figure 1:**
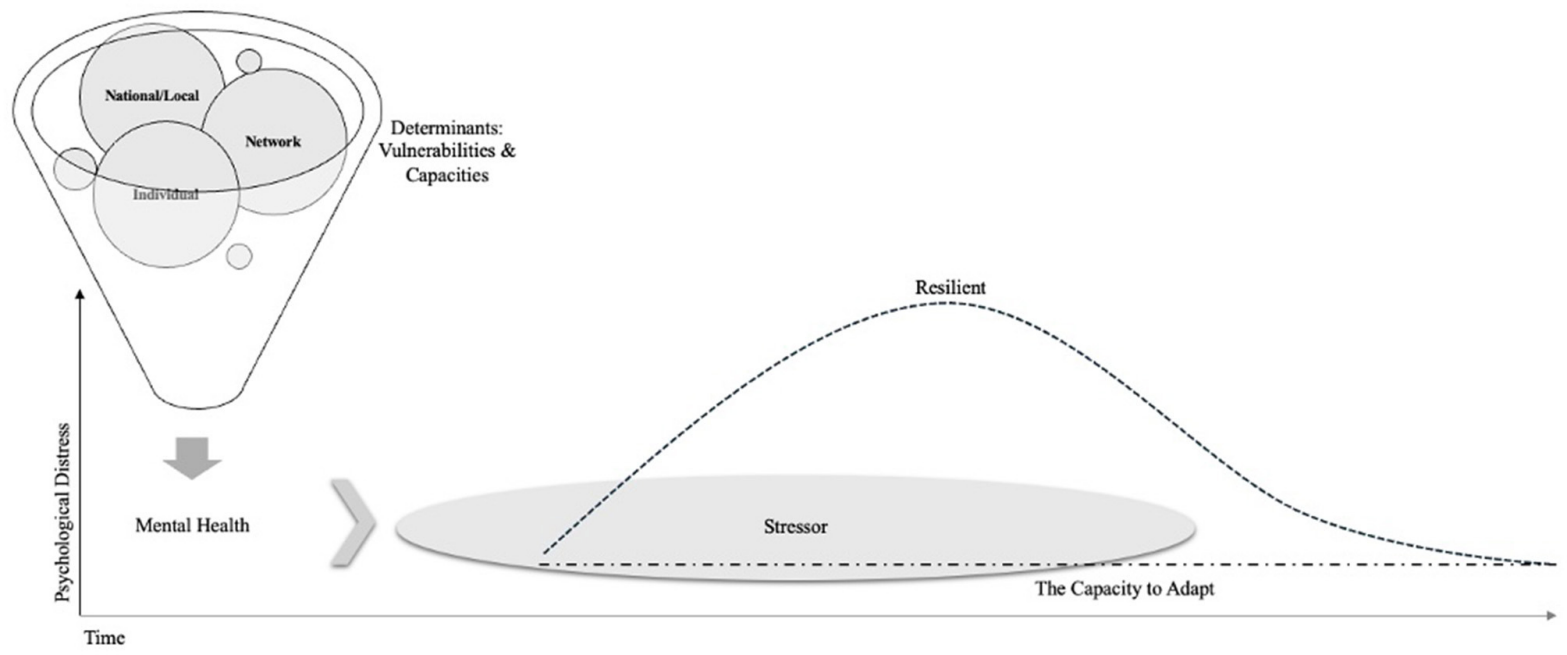
A model of resilience and adaptability.

A second often cited definition of resilience frames it as an indicator of adaptability in the face of ongoing adversity (Schetter and Dolbier, 2011; Southwick et al., 2014). In other words, this definition conceptualizes individuals as resilient when they demonstrate a consistent state of stability in the context of adversity or chronic stress. Such individuals do not exhibit the transient increase in distress implicated within the first definition of resilience and instead exhibit an ability to adapt to the pressures of their environment (Rudenstine et al., 2022). Given this unique emphasis on adaptation, we propose referring to this second definition, not as resilience, but as what it is: the capacity to adapt (Figure 1). In contexts where stressors are inevitable, whether time-limited or chronic, a segment of the population can sustain a baseline level of psychological health. Adaptability is acclimating and adjusting while maintaining healthy functioning amidst stressors.

Resilience and adaptability differ in their contribution to symptom progression and align with different prevention strategies. For example, when focusing on resilience—a trajectory of distress and recovery—the primary focus is on individuals and communities exhibiting signs of acute distress or disorder (re)occurrence to determine what fosters recovery. On the other hand, when focusing on adaptability, the key focus is on those factors that protect against the initial onset of distress in the context of ongoing stress exposure. Moreover, being adaptable is not synonymous with experiencing posttraumatic growth as the latter refers to shifts that occur due to the stressors experienced while adaptability refers simply to the maintenance of baseline psychological functioning. The factors that contribute to adaptive capacity, therefore, are worthy targets for prevention.

## Defying risk: the capacity to adapt as key to primary prevention

The growing national and global mental health crisis warrants a renewed call to shift financial investment from treatment to prevention. While this call to action is not new, prevention efforts remain underfunded (Rudenstine and Galea, 2015). There are two leading frameworks for prevention, each using a three-tiered conceptualization of prevention targets [Institute of Medicine (US) Committee on Prevention of Mental Disorders, 1994]. Despite these frameworks, the field of mental health has yet to mobilize around a strategy for action.

By attenuating risk factors it is possible to reduce risk for new onset and/or the progression of mental health problems. Informed by the nuanced etiologies of mental illness we suggest a framework that targets factors associated with both the risk of, and protection against, mental disorders at the individual, network, and local/national levels. In addition, defining early life as a sensitive period for mental health we consider those factors that are best developed in childhood as well as the contexts in which primary prevention efforts would be most effective if established (Knudsen, 2004).

During early life, 0–5 years old, there are significant and rapid changes to the brain that allow for learning and emotional stability (Shonkoff et al., 2000). The presence of reliable and nurturing caregivers sets the stage for secure attachment in children, which is the bedrock to emotional and interpersonal health (Mikulincer and Shaver, 2012). A correlate of attachment, experiencing agency and an internal locus of control, is a documented buffer for negative psychological outcomes in the context of adversity (Kesavayuth et al., 2022). Emotion regulation, emotional intelligence, and mentalization have each been found to evolve out of a child's secure attachment to a caregiver and contribute to one's ability to metabolize affective experiences (Rosso, 2022; Weinberg, 2006). In this vein, the case for adaptability is made by the ever-growing body of literature that has found these affective capacities to mitigate the negative mental health outcomes associated with adversity (Ballespí et al., 2021; Ciarrochi et al., 2002; Davis and Humphrey, 2012; Hu et al., 2014; Naragon-Gainey et al., 2017; Rudenstine and Espinosa, 2018).

### Individual level

A number of individual-level factors are known to contribute to adaptability. Primary prevention at the individual level includes programming that fosters developmental psychological abilities widely known to promote psychological health throughout the life course. For example, *Parents First* provides parental support that fosters healthy and sustaining parent-child relationships by developing parents' reflective functioning, which is highly correlated with positive outcomes in children (Slade, 2007). Similarly, instituting skin-to-skin contact between mothers and newborns, a zero-cost intervention, has meaningful physiological outcomes and is associated with secure attachment between infants and mothers (Conde-Agudelo et al., 2011; Moore et al., 2012; Redshaw et al., 2014). Outside the family system, school-based programs that focus on social-emotional learning and stress management skills have demonstrated effective promotion of mental wellbeing among children and adolescents as these capacities buffer the negative mental health outcomes associated with adversity and stress (Weissberg, 2019). Related, given the wealth of data showing the protective role of emotional intelligence and emotion regulation against psychiatric disorders, clinical interventions—such as Dialectical Behavior Therapy—are being adapted and implemented in clinical and non-clinical settings (Harvey et al., 2023; Mazza et al., 2016). While there is indisputable value in implementing programming that fosters individual-level factors throughout the life course, investing specifically in the development of these capacities in early life has the potential to inoculate individuals against negative mental health outcomes by equipping them with the capacity to adapt when faced with inevitable future stressors.

### Network level

Engaging stakeholders is a fundamental step when introducing mental health prevention efforts or intervention services to new communities (Duncan et al., 2023; Walker et al., 2018). This collaboration increases the likelihood that that the programming will address the needs and cultural context of the community, which in turn will increase engagement by the community as well as their effectiveness. In addition, the process of involving stakeholders, including people with lived experience, local politicians, religious leaders, school officials and local healthcare workers, helps to make the new efforts sustainable. In this way, partnerships with known and trusted community and school-based organizations engage individuals in familiar, supportive environments, which can increase access to services, counter stigma around help-seeking, and intuit acceptance of interventions. Participant based research provides a framework for how to successfully work individuals with direct experience and knowledge of an issue and the affected population (Baum et al., 2006).

Infusing schools, especially preschools and elementary schools, with curriculum and programs that foster adaptability have broad reach and provide essential tools to children during a sensitive developmental period (O'Reilly et al., 2018). Youth groups and clubs that increase community bonds and support, parenting classes, and education programs to prevent substance abuse are other examples of primary prevention that target youth via the environments they spend most time in (Panchal et al., 2022). For adults, workplace initiatives, including stress reduction programs, mental health awareness training, and supportive organizational policies, similarly have been found to reduce mental health issues by raising awareness and promoting healthy routines. These interventions result in increased productivity and reduced absenteeism. Integrating programming that fosters mental health within everyday settings combats stigma associated with mental illness and help-seeking, fosters engagement, and is more likely to be culturally attuned. While such programming requires financial investment and political capital, there are examples of how such efforts have been integrated into youth programming (including schools) as well as guidelines from the US Department of Labor for employers committed to fostering mental health of their employees (Greenberg, 2023; US Department of Labor, 2025).

### Local and national level

Mental health prevention at a local and national level involves implementing strategies that address the social, structural, and individual determinants of mental health to reduce risks, build resilience, and foster supportive environments. Effective policies require a multisectoral approach, engaging education, labor, justice, housing, and welfare alongside health services (Castillo et al., 2019; Grummitt et al., 2023; Kirkbride et al., 2024). To date, such policies often seek to address inequities with the goal of reducing persistent stressors, such as food insecurity, that increase one's risk for mental health outcomes. Comprehensive strategies include promoting mental health literacy, integrating mental health into general healthcare systems, increased funding to enable public and private organizations integrate mental health access into their systems, and developing free and easily accessible resource centers that assist individuals in accessing resources (Campion et al., 2022; Hodgkinson et al., 2017; McGinty and Daumit, 2020; Purtle et al., 2020; Barry et al., 2023).

Over the past decade, technology has become critical to the dissemination of information and in reducing barriers to care. At the national level in primary prevention for mental health technology offers accessible, scalable, and innovative ways to promote wellbeing and prevent the onset of mental health issues (Burns and Birrell, 2014). Mobile apps, web-based programs, and online platforms are all used to make psychoeducation, emotional learning, and stress management tools widely available (Singh et al., 2022). In addition, these technologies have been found to aid early detection and screening of mental health risks (Liu et al., 2023). Digital platforms that are anonymous reduce stigma associated with seeking mental health support as individuals are able to participate without fear of judgment.

No single factor can fully protect against negative mental health outcomes, rather the presence of various factors jointly contribute to adaptability. Therefore, primary prevention across these three levels requires a coordinated effort involving caregivers, educators, healthcare providers, policymakers, employers, and community leaders. Even more, it requires that governing agencies and corporations choose to invest proactively in the psychological wellbeing of the population by fostering those factors that are widely known to buffer the negative consequences of life stressors.

## Conclusion

The rising prevalence of mental disorders suggests that current strategies are inadequate, necessitating a shift toward proactively building adaptability to address this growing public health concern. By expanding our focus beyond resilience as mere recovery to emphasizing adaptability as the capacity to maintain wellbeing amid chronic stress, we can identify targets for primary prevention that will reduce the incidence of mental illness. A three-pronged approach that emphasizes the importance of early life experiences, targets individual psychological capacities, strengthens support networks, and implements supportive local and national policies and programs represents a promising path forward.

To actualize the framework of adaptability as a cornerstone of primary prevention, future research and implementation efforts must move toward measurable, multi-level operationalization. At the individual level, longitudinal studies should assess how early interventions targeting such phenomenon as emotion regulation, attachment security, internal locus of control, and self-esteem predict long-term mental health outcomes in the context of chronic stress. Experimental and quasi-experimental designs can evaluate which combinations of early life interventions (e.g., reflective parenting programs, school-based emotional learning) most effectively cultivate adaptive capacity across developmental stages. In these investigations, it will be critical to consider how the notion of adaptability varies across cultural contexts and if the building blocks for the capacity to adapt vary across cultures. At the network level, participatory research that partners with schools, faith institutions, and local organizations can examine how culturally responsive, community-embedded programs foster adaptability in high-risk populations. Implementation science should be employed to identify barriers and facilitators to sustaining such programs across diverse contexts. At the local and national levels, policy-driven research should evaluate the population-wide mental health effect of multisectoral investments in structural supports—such as food security, stable housing, and digital access to psychoeducational tools—designed to reduce persistent stressors. Critically, the adaptability framework calls for the development of new metrics capable of capturing adaptive functioning (e.g., sustained emotional stability, engagement in social roles) in the context of inevitable stress exposure over the life course. Across these various levels, incorporating data privacy protocols will be essential. Such precautions are critical to prevention efforts as such programming often monitors sensitive personal data, including trauma history, stress levels, behavioral patterns, and sociodemographic factors. As a result, population-level interventions may involve very large data banks of such sensitive information. There have been meaningful developments in the area of data privacy that can offer clear recommendations for action (Lustgarten et al., 2020; Zhang et al., 2025).

Several limitations warrant consideration. First, adaptability is not yet a standardized construct in mental health research; developing valid and reliable measures for adaptability is critical. Second, the framework risks overemphasizing individual-level responsibility in contexts where structural inequities are the dominant drivers of chronic stress. In doing this, the framework seeks to identify a path for equipping individuals to navigate inevitable life stressors. Third, while adaptability is positioned as a protective factor, it may not be equally attainable or beneficial across all populations, as it needs to be evaluated in the presence and absence of fundamental resources and supports. Fourth, this paper is informed by a careful review of the literature on resilience and adaptability; a systematic review that documents the factors that contribute to resilience and adaptability as well as how these factors may vary in relevance across cultures is an important next step to inform research, practice, a policy for adaptability. Advancing this agenda will require a paradigm shift in how we define, measure, and invest in mental health prevention—moving from episodic crisis response toward equipping individuals and communities to thrive amid life's inevitabilities.

In this way, by centering our prevention efforts on cultivating adaptability, we move beyond reactive interventions and foster proactive strategies that empower individuals and communities to thrive in the face of adversity. This approach acknowledges the inevitability of lifetime stressors and seeks to equip individuals to navigate such events while maintaining their baseline mental health. Such a shift requires financial investment in interventions that, if successful, has the potential to be measurable not at the individual level, but through a gradual reduction in the incidence of mental disorders across the population. While this long-term investment makes adaptability hard to measure (or “prove”), incorporating adaptability into the broader prevention discourse has may lead to healthier individuals and communities and be associated with societal and economic benefits.
